# Early Collaboration Measures Among a Cross-Sector Stakeholder Group in a Statewide GWEP

**DOI:** 10.1093/geroni/igab046.2815

**Published:** 2021-12-17

**Authors:** Jennifer Crittenden, Abigail Elwell, David Wihry, Lenard Kaye

**Affiliations:** 1 University of Maine, University of Maine, Maine, United States; 2 University of Maine, Bangor, Maine, United States

## Abstract

The AgingME Geriatrics Workforce Enhancement Program (GWEP) uses collaboration across institutions of higher education, community-based organizations, and healthcare entities to imbed transformational healthcare practice change across Maine, a primarily rural state. To explore the factors that influence cross-sector collaboration among a diverse array of partners, a baseline anonymous electronic survey was distributed to the newly formed project steering committee. The survey consisted of the Wilder Collaborative Factors Inventory, an established measure of 22 research-based collaboration factors along with four open response questions on process-level challenges and opportunities for improvement. A total of eleven responses (N = 11) were received out of 20 Steering Committee members (55% response). Collaboration strengths noted in the assessment include unique purpose of statewide GWEP efforts (M = 4.41 out of 5 points), mutual trust among members (M = 4.32), favorable social and political environment (M = 4.27), and a history of collaboration among partners (M = 4.27). Lower scores were received on the multiple layers of participation (M = 3.45 out of 5 points), and ability to compromise factors (M = 3.45). Qualitative responses reinforced the need for a common understanding of the project’s goals and outcomes early on in the collaboration. Barriers to collaboration included scheduling considerations and limited time and energy among partners due to heightened COVID-19 response efforts. Results elucidate: 1) Early collaboration strengths and needs of a newly formed statewide education collaborative; and 2) Strategic action steps and focal points informing early partnershipping among organizations engaged in interprofessional health education efforts.

